# Antiosteoporosis Activity of New Oriental Medicine Preparation (Kyungokgo Mixed with Water Extract of *Hovenia dulcis*) on the Ovariectomized Mice

**DOI:** 10.1155/2015/373145

**Published:** 2015-02-09

**Authors:** Yun-Ho Hwang, Kwang-Jin Kim, Jong-Jin Kim, Kyung-Yun Kang, Sung-Ju Lee, Gil-Yeon Jeong, Kyung-Hee Choi, Young-Jin Son, Sung-Tae Yee

**Affiliations:** ^1^Department of Pharmacy, Sunchon National University, Suncheon 540-742, Republic of Korea; ^2^Department of Biology, Sunchon National University, Suncheon 540-742, Republic of Korea

## Abstract

Protective effect of new oriental medicine (Kyungokgo mixed with water extract of *Hovenia dulcis*, KOGHD) was assessed on the bone loss induced mice by ovariectomy. In the *in vivo* experiments, antiosteoporosis effect of KOGHD was investigated using ovariectomized osteoporosis mice model. After 6 weeks of treatment, the mice were euthanized, and the effect of Kyungokgo (KOG) and KOGHD on body weight, spleen weigh, thymus weight, uterine weight, serum biochemical indicators, bone weight and length, immune cell population, bone morphometric parameters, and histological stains was observed. Our results showed that KOGHD prevented the deterioration of trabecular microarchitecture caused by ovariectomy, which were accompanied by the lower levels of bone turnover markers and immune cell population as evidenced by the inhibition of RANKL-mediated osteoclast differentiation without cytotoxic effect on bone marrow derived macrophages (BMMs). Therefore, these results suggest that the *Hovenia dulcis* (HD) supplementation in the KOG may also prevent and treat bone loss.

## 1. Introduction

Osteoporosis is a major public health problem that mainly affects middle-aged and elderly women. It is characterized by deterioration of bone microarchitecture and the loss of bone mass, which increase bone fragility and the risk of fracture. These symptoms have been observed in menopausal or postmenopausal women and are associated with reduced levels of estrogen [1–3]. In particular, estrogen deficiency in postmenopausal women increases the rate of bone remodeling by causing an imbalance between activities of osteoblasts and osteoclasts in bone, which can lead to fracture or inflammatory diseases, such as osteoporosis, rheumatoid arthritis, systemic lupus erythematous, inflammatory bowel disease, and chronic obstructive pulmonary disease [4–6]. Osteoporosis is caused by decreased estrogen levels in ovaries and can be prevented by estrogen replacement therapy (ERT) or hormone replacement therapy (HRT). The agents currently used to treat osteoporosis are antiresorptive agents, such as estrogen, bisphosphonates, raloxifene, and calcitonin. However, ERT/HRT has many side effects, such as breast cancer, endometrial cancer, heart attacks, strokes, and blood clots [7]. Therefore, it is necessary to develop alternative medicine with less undesirable side effects that reduced the need for the drugs used currently.

Estrogen deficiency in postmenopausal osteoporosis, following ovariectomy, enhances the rate of bone resorption by increasing osteoclast formation from bone marrow derived macrophages (BMM), as induced by macrophage colony stimulating factor (M-CSF) and a TNF-related factor known as receptor activator of NF-*κ*B ligand (RANKL). The productions of M-CSF and RANKL are known to be induced by the ovariectomy-enhanced T cell production of TNF-a. In other words, T cells play an important role in ovariectomy-induced bone loss [8].

Kyungokgo (KOG) is composed of* Panax ginseng*,* Poria cocas* Wolf,* Rehmannia glutinosa* Liboschitz var.* purpurea,* and honey; it is used to treat general weakness in traditional oriental medicine. Furthermore, KOG is known to contain amino acids and minerals and to have an antioxidant effect [9–11]. Some studies have reported that KOG has beneficial effects on hyperglycemia, hypertension, fatigue, inflammation, and gastric ulcer [12, 13], and, interestingly, a water extract of KOG was found to have a proliferative effect on osteoblasts without cytotoxic effect* in vitro* and to decrease the bone loss induced by LPS* in vivo* [14].

*Hovenia dulcis* Thunb. (Rhamnaceae), also known as the Japanese raisin tree, is commonly found in East Asia (China, Japan, Korea, and India), and its leaves, seeds, fruit, roots, and bark are used as traditional herbal medicines [15]. Recent studies have demonstrated that polysaccharides from the peduncles of* H. dulcis* have a significant hepatoprotective effect on acute alcohol-induced liver injury in mice via their antioxidant activities [16]. In another study, it was demonstrated that phenolic compounds from* H. dulcis* had protective effects on glutamate-induced neurotoxicity in HT22 cells and that these effects were due to their free radical scavenging activities [17].

Despite many reports concerning the effects of KOG and HD, no examination of the antiosteoporotic effects of KOG or KOGHD on postmenopausal osteoporosis was induced by ovariectomy (OVX) in mice. We hypothesized that KOGHD may beneficially prevent bone loss induced by estrogen deficiency. In the present study, we attempted to demonstrate the beneficial effects of KOGHD on RANKL-induced osteoclast differentiation as well as the fact that the inhibitory effects of KOGHD on the bone deterioration were examined to verify the value of KOGHD as a preventing agent for mediating bone diseases.

## 2. Materials and Methods

### 2.1. Preparation of Sample

The KOG (kyungokgo), KOGHD10% (kyungokgo + 10%* Hovenia dulcis* extracts), and KOGHD25% (kyungokgo + 25%* Hovenia dulcis*) were provided by a JBT company. KOG was made in the following way.* Ginseng* (7.8%) and* Poria cocos* (15.6%) were mixed, and the dried juice of* Rehmannia glutinosa* (25.6%) and honey (51%) was added. This mixture was heated in boiling water for 72 hours, cooled for 24 hours, and boiled for a further 24 hours. KOGHD10% and KOGHD25% were made in the same way as KOG using the following drug ratios.

KOGHD10%:* Ginseng* (7.8%) +* Poriacocos* (15.6%) +* Rehmannia glutinosa* (15.6%) + honey (51%) +* Hovenia dulcis* extracts (10%).

KOGHD25%:* Ginseng* (7.8%) +* Poria cocos* (15.6%) +* Rehmannia glutinosa* (0.6%) + honey (51%) +* Hovenia dulcis* extracts (25%).

### 2.2. Reagents

Recombinant mouse/human RANKL and macrophage-colony stimulating factor (M-CSF) were purchased from R&D Systems (MN, USA). Cell culture medium, fetal bovine serum (FBS), penicillin, and streptomycin were purchased from Invitrogen Life Technologies (CA, USA). The CCK-8 assay kit was obtained from Dojindo Molecular Technologies (Japan).

### 2.3. Cell Cultures and Osteoclast Differentiation

Bone marrow cells (BMCs) were obtained from femurs and tibiae of 5- to 6-week-old ICR mice after sacrifice by cervical dislocation. BMCs were cultured with M-CSF (10 ng/mL) for 1 day, and nonadherent cells were cultured for further 3 days in the presence of M-CSF (30 ng/mL). Adherent cells (BMMs) were isolated and induced with RANKL (10 ng/mL) in the presence of M-CSF (30 ng/mL) for 4 days. Osteoclastogenesis was determined by TRAP staining according to the manufacturer's instructions (Sigma-Aldrich, MO, USA).

### 2.4. TRAP Staining

The cultured cells were fixed with 3.7% formalin for 5 min and permeabilized with 0.1% Triton X-100 for 5 min. Cells were then stained with TRAP solution (Sigma-Aldrich, MO, USA) for 10 min. TRAP-positive multinuclear cells (nuclei ≥ 3) were counted as osteoclasts.

### 2.5. Measurement of TRAP Activity

TRAP-stained cells were fixed with 3.7% formalin for 5 min, permeabilized with 0.1% Triton X-100 for 5 min, and treated with TRAP buffer (100 mM sodium citrate pH 5.0, 50 mM sodium tartrate) containing 3 mM p-nitrophenyl phosphate (Sigma-Aldrich, MO, USA) at 37°C for 5 min. Reaction mixtures were transferred into new plates containing an equal volume of 0.1 N NaOH, and optical densities (OD) were measured at 405 nm.

### 2.6. Cytotoxicity Assay

BMMs were cultured at a density of 1 × 10^4^ cells/well on 96-well plates in triplicate with M-CSF (30 ng/mL) and HD extract. After 3 days, cells were treated with CCK-8 reagent and incubated for 4 hours, according to the manufacturer's protocol (Dojindo Molecular Technologies, Japan). Absorbance was read on an ELISA reader at 450 nm.

### 2.7. Animals and Experimental Treatments

Female C3H/HeN mice (8-week-old) and ICR mice (5 to 6-week-old), weighing 20–22 g, were purchased from Orientbio (Orientbio Inc, Iksan, Korea). All mice were treated in strict accordance with Sunchon National University Institutional Animal Care and Use Committee (SCNU IACUC) guidelines for the care and use of laboratory animals (Permit Number: 2013-9). All surgery was performed under zoletil anesthesia, and all efforts were made to minimize suffering. All experimental procedures were reviewed and approved by the SCNU IACUC. Animals were housed in standard polycarbonate cages in a controlled environment (22 ± 2°C, RH 50–60%) under a 12 h light/dark cycle and supplied commercial rodent chow (DAE-HAN Biolink, Deajeon, Korea) and water* ad libitum*. Mice were randomly divided into 6 groups of 5 animals:

 SHAM: sham control mice treated with vehicle;
 OVX: bilaterally ovariectomized control mice treated with vehicle;
 
*E*_2_: OVX mice treated with *β*-estradiol water soluble (0.03 *μ*g/head/day, s.c);
 KOG: OVX mice treated with KOG (185 mg/kg);
 KOGHD10%: OVX mice treated with KOGHD10% (185 mg/kg);
 KOGHD25%: OVX mice treated with KOGHD25% (185 mg/kg).

All mice were bilaterally ovariectomized after inducing zoletil anesthesia. Vehicle (sterilized distilled water) or a sample (KOG, KOGHD10%, or KOGHD25%) was orally administered daily for 6 weeks from one day after surgery.

### 2.8. Weight and Length Measurements

At the end of treatments, final body weights were recorded. Animals were sacrificed by cervical dislocation on scheduled dates and weights of organs (uterus, spleen, and thymus) were recorded by using electronic scale. The weights and lengths of isolated right femurs and tibiae were measured. Femur length was defined as the maximal distance between the head of the greater trochanter and the distal condyles, and tibia length was defined as the maximal distance between proximal condyles and the malleolus. All measurements were made using a vernier caliper. Weights were measured immediately after lengths.

### 2.9. Estimation of Serum Ca, IP, ALP, and TCHO

Blood was collected from the retroorbital region to assess biochemical parameters and centrifuged at 5000 rpm for 5 min. Serum was separated immediately and stored at −20°C. Serum calcium (Ca), inorganic phosphorus (IP), alkaline phosphatase (ALP), and total cholesterol (TCHO) levels were measured by an automatic analyser (Fuji Dri-Chem, Fuji, Japan) using a diagnostic slide kit.

### 2.10. Measurements of TRAP and *E*_2_ in Sera by ELISA

Serum estradiol (*E*_2_) and circulating markers of bone resorption (tartrate-resistant acid phosphatase, TRAP) levels were measured using an estradiol enzyme-linked immunoassay (ELISA) kit (Calbiotech, San Diego, CA, USA) or a TRAP ELISA kit (USCN Life Science, Wuhan, P.R. China). All analyses were performed according to protocols provided by the manufacturers.

### 2.11. Bone Microarchitecture Analysis by Micro-CT

Morphological measurements, including bone volume density (BV/TV), BS/TV, BS/BV, trabecular thickness/separation/number/pattern factor (Tb.Th, Tb.Sp, Tb.N, Tb.Pf), and structure model index (SMI) were calculated from microcomputed tomography (micro-CT) data for each mice using Skyscan 1172 (Skyscan, Kontich, Belgium). Regions of interest for analysis were the proximal tibia and distal femur metaphysis. User-defined contours were outlined on every fifth slice of a 150-slice region extending 2.5 mm distally from the growth plate, starting at the point where growth plate tissue was no longer visible in the grayscale CT slice. For quantification of trabecular bone mineral density (BMD), the micro-CT was calibrated using two standard phantoms with a density of 0.25 and 0.75 g/cm^2^. The image slices were reconstructed and analysed using CTAn analyser software (Skysacn) [18].

### 2.12. Immunophenotyping of Lymphocytes

To identify immunophenotype of lymphocyte, immune cells were isolated from spleen and thymus. In order to remove red blood cells, blood was treated with BRC lysis buffer for one minute. Aliquots of 1 × 10^6^ lymphocytes treated with a blocking anti-FcR (BD Pharmingen) were stained with FITC anti-CD4 or CD19, PE anti-CD8 or CD11c mAb for 30 min at 4°C. Cells were then washed with FACS solution buffer (PBSx1 + 10% FBS + 1% Na azide). Lymphocytes were gated and analyzed on a BD FACS Canto II flow cytometer (BD Biosciences).

### 2.13. Histological Analysis

Tibiae were fixed in 4% paraformaldehyde, decalcified in 10% EDTA, dehydrated, embedded in paraffin, sectioned at 5 *μ*m, and stained with hematoxylin and eosin (H&E). TRAP staining was used to determine osteoclast activity. Deparaffinized sections were added to TRAP reagent, which contained 225 *μ*M naphthol AS-MX phosphate (Sigma-Aldrich, St. Louis, MO, USA), 0.84% N, N-dimethylformamide (Sigma-Aldrich), and 1.33 mMFast Red Violet LB Salt (Sigma-Aldrich) in 50 mM sodium acetate (pH 5.0) containing 50 mM sodium tartrate and incubated for 1 h. After incubation, sections were washed in distilled water and counterstained with 1% methyl green. The region of tibiae studied was the secondary spongiosa, the trabecular portion of proximal tibiae at 12 mm distal to the epiphyseal plate and extending to 6 mm. Sections (7 mm) were deplasticized in 2-ethoxyethyl acetate and stained with Masson trichrome.

### 2.14. Statistical Analysis

Differences in data between groups are presented as the mean ± S.D. of 3 replicates. Statistical differences were analyzed using Student's* t*-test. Probability values less than 0.05 were considered significant (*P* values ^*^<0.05, ^**^<0.01, ^***^<0.001).

## 3. Results

### 3.1. HD Suppressed Osteoclast Differentiation from BMMs and Had No Cytotoxic Effect

**Figure 1:**
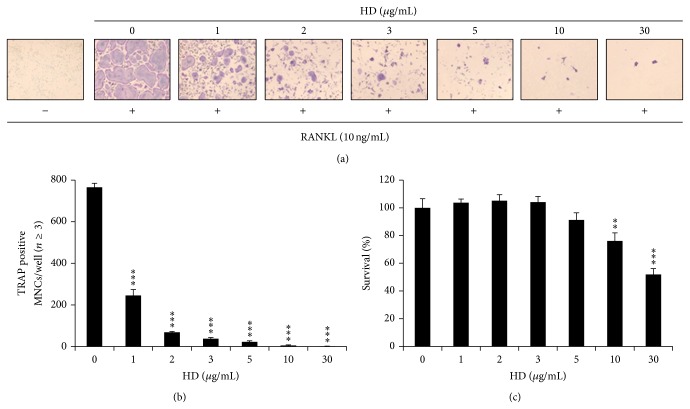
HD suppresses osteoclastogenesis. (a) BMMs prepared from bone marrow cells were cultured for 4 days with RANKL (10 ng/mL) and M-CSF (30 ng/mL) in the presence of the indicated concentrations of HD. Cells were fixed in 3.7% formalin, permeabilized in 0.1% Triton X-100, and stained for TRAP, an enzyme marker of osteoclasts. (b) TRAP-positive multinuclear cells with three or more nuclei were counted as osteoclasts. (c) Effect of HD on the viability on BMMs was evaluated by CCK-8 assay. ^***^ *P* < 0.005.

To examine whether HD inhibits RANKL-induced osteoclast differentiation, BMMs were cultured with RANKL (10 ng/mL) and M-CSF (30 ng/mL) in the presence of HD for 4 days. RANKL induced the formation of TRAP-positive osteoclasts, but HD suppressed RANKL-induced TRAP-positive osteoclast differentiation (Figure 1(a)). Furthermore, the number of osteoclasts was significantly and dose-dependently decreased by HD (Figure 1(b)). These results suggest that HD can inhibit RANKL-mediated osteoclast differentiation. To exclude the possibility that HD inhibits osteoclast differentiation in this manner. BMMs were incubated without RANKL in the presence of M-CSF (30 ng/mL) and HD. No difference in the survival rates of cells cultured was observed at HD concentrations < 5 *μ*g/mL (Figure 1(c)). These results suggest that lower concentrations HD is not toxic to BMMs and that it only inhibits osteoclastogenesis.

### 3.2. Effects of HD on the Trabecular Bone Microarchitectural Changes

**Figure 2:**
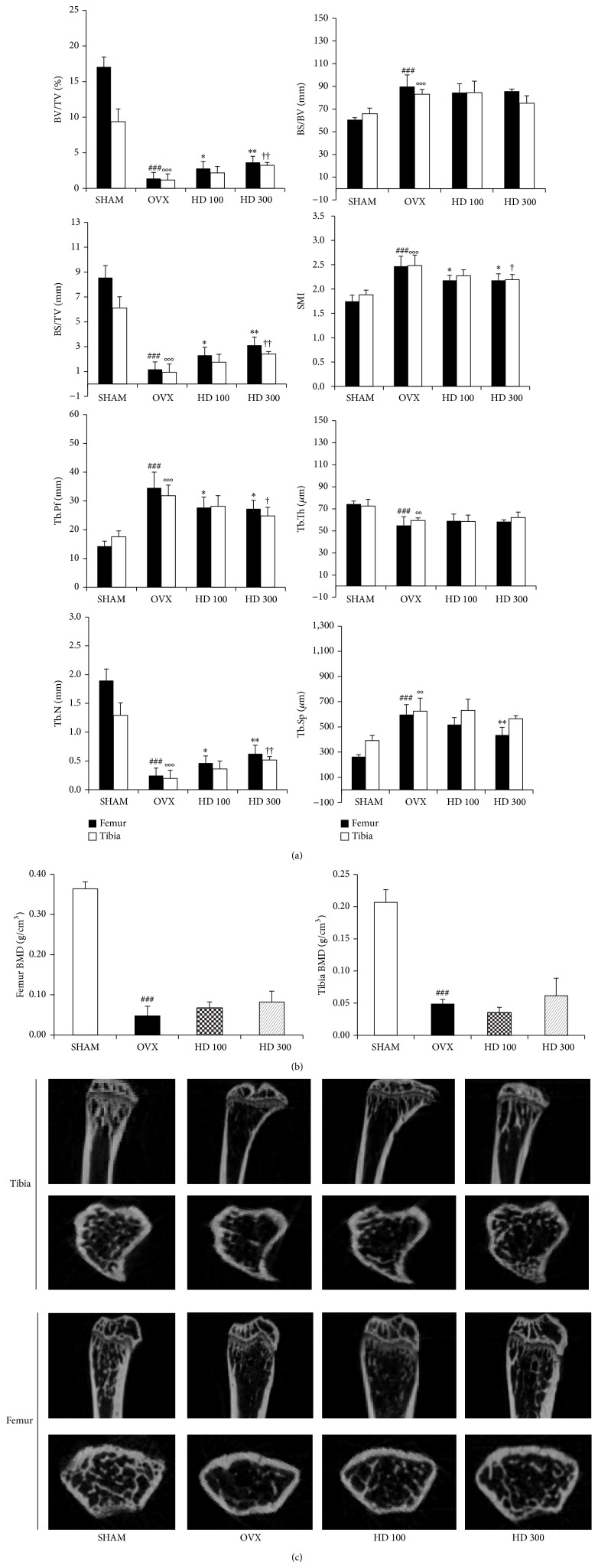
Effect of HD (*Hovenia dulsis*) on trabecular morphometric parameters in proximal tibia and distal femur of OVX mice. Mice were treated with vehicle, HD (100, 300 mg/kg/day, p.o) for 6 weeks. (a) Bone volume/tissue volume (BV/TV), bone surface/tissue volume (BS/TV), bone surface/Bone volume (BS/BV), structure model index (SMI), trabecular bone pattern factor (Tb.Pf), trabecular thickness (Tb.Th), trabecular number (Tb.N), and trabecular separation (Tb.Sp) as analyzed with micro-CT Skyscan CTAn software. Data are expressed as mean SD (*n* = 5). ^*^ *P* < 0.05, ^**^ *P* < 0.01, and ^***^ *P* < 0.001 versus OVX group (femur); ^#^ *P* < 0.05, ^##^ *P* < 0.01, and ^###^ *P* < 0.001 versus sham group (femur); ^†^ *P* < 0.05, ^††^ *P* < 0.01, and ^†††^ *P* < 0.001 versus OVX group (tibia); °*P* < 0.05, °°*P* < 0.01, and °°°*P* < 0.001 versus sham group (tibia). (b) Effect of HD on BMD of femur and tibia of OVX mice. Data are expressed as means SD (*n* = 5). ^*^ *P* < 0.05, ^**^ *P* < 0.01, and ^***^ *P* < 0.001 versus OVX group; ^#^ *P* < 0.05, ^##^ *P* < 0.01, and ^###^ *P* < 0.001 versus sham group. (c) Representative two-dimensional images of the tibia and femur in the SHAM, OVX, HD 100, and HD 300 groups.

To determine protective effects of HD on the bone loss induced by estrogen deficiency, bone microarchitectural changes were analyzed by micro-CT. In proximal tibia and distal femur, BV/TV (bone volume/total volume), BS/TV (bone surface/total volume), and Tb.N (trabecular number) were increased in HD group than in OVX group. The SMI (structure model index) and Tb.Pf (trabecular pattern factor) were decreased in HD group as compared with the OVX group. However, BS/BV (bone surface/bone volume) and Tb.Th (trabecular thickness) in HD group were not changed when compared to the OVX group. In distal femur, Tb.Sp (trabecular separation) in HD group was lower than in OVX (Figure 2(a)). Trabecular BMD (bone mineral density) of tibia and femur was decreased in OVX group when compared to the SHAM group. However, HD did not restore BMD to the level in the SHAM group (Figure 2(b)). Two-dimensional images of proximal tibiae and distal femoral metaphyses showed that bone loss in OVX mice was slightly inhibited by the oral administration of HD (Figure 2(c)).

### 3.3. KOGHD Suppressed Osteoclast Differentiation and Had No Cytotoxic Effect on BMMs

**Figure 3:**
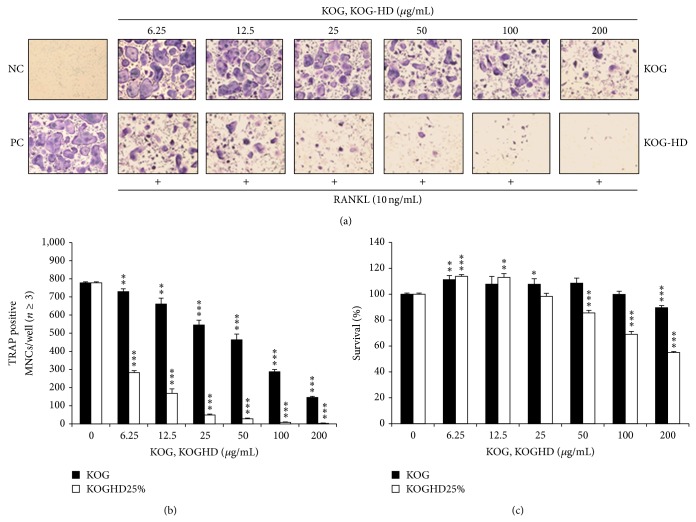
KOGHD suppresses osteoclastogenesis. (a) BMMs prepared from bone marrow cells were cultured for 4 days with RANKL (10 ng/mL) and M-CSF (30 ng/mL) in the presence of the indicated concentrations of KOG and KOGHD. Cells were fixed in 3.7% formalin, permeabilized in 0.1% Triton X-100, and stained for TRAP, an enzyme marker of osteoclasts. (b) TRAP-positive multinuclear cells with three or more nuclei were counted as osteoclasts. (c) Effect of KOG-HD on the viability on BMMs was evaluated by CCK-8 assay. ^***^ *P* < 0.005.

To examine whether KOGHD inhibits RANKL-induced osteoclast differentiation, BMMs were cultured with RANKL (10 ng/mL) and M-CSF (30 ng/mL) in the presence of KOGHD for 4 days. KOG was found to have a slight inhibitory effect on osteoclast differentiation, whereas KOGHD considerably suppressed RANKL-induced TRAP-positive osteoclast differentiation (Figure 3(a)). Furthermore, the inhibitory effect of KOGHD on osteoclast differentiation was not due to its cytotoxicity (Figures 3(b) and 3(c)). These results suggest that HD reinforced the inhibitory effect of KOG against RANKL-mediated osteoclast differentiation.

### 3.4. Effects of KOG, KOGHD10% and KOGHD25% on Body Weights, Bone and Organ Weights (Uterus, Spleen, Thymus) in OVX Mice

**Figure 4:**
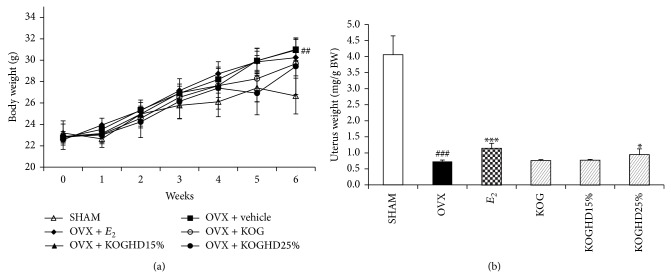
Effect of KOG, KOGHD10%, and KOGHD25% on (a) body weight and (b) uterus in OVX mice. They were treated with vehicle, sample (185 mg/kg/day, p.o) for 6 weeks. Data are expressed as means ± SD, *n* = 5. ^*^ *P* < 0.05, ^**^ *P* < 0.01, and ^***^ *P* < 0.001 versus OVX group; ^#^ *P* < 0.05, ^##^ *P* < 0.01, and ^###^ *P* < 0.001 versus SHAM group.

The experimental animals in the SHAM, OVX, KOG, KOGHD10%, and KOGHD25% groups had similar initial mean body weights. After treatment for 6 weeks, body weight and spleen weight gains in the OVX group were significantly greater than in the SHAM group. Body weights in KOG, KOGHD10%, and KOGHD25% group were not suppressed to SHAM group levels (Figure 4(a)). Spleen weight in the KOGHD25% group was lower than in the OVX group. The thymus weight in OVX group was slightly increased, but this was not statistically significant (Table 1). Uterus weights in OVX group were lower than in the SHAM group (*P* < 0.01). This was attributed to uterine atrophy caused by the lack of an estrogen supply from ovaries. However, uterus weights in KOGHD25% group (185 mg/kg BW of KOGHD25%) were significantly greater than in the OVX group (Figure 4(b)). Lengths of tibiae and femurs were not significantly different between the study groups. However, bone weights were significantly different. Mean weights of tibiae in the KOGHD10% (0.048 g ± 0.001) and KOGHD25% (0.05 g ± 0.002) groups were significantly higher than in the OVX group (0.044 g ± 0.002). Furthermore, mean femur weights were higher in the in KOGHD10% (0.063 g ± 0.001) and the KOGHD25% (0.065 g ± 0.001) groups than in the OVX group (0.058 g ± 0.003) (Table 2).

### 3.5. Effects of KOG, KOGHD10%, and KOGHD25% on Serum Biochemical Markers

**Figure 5:**
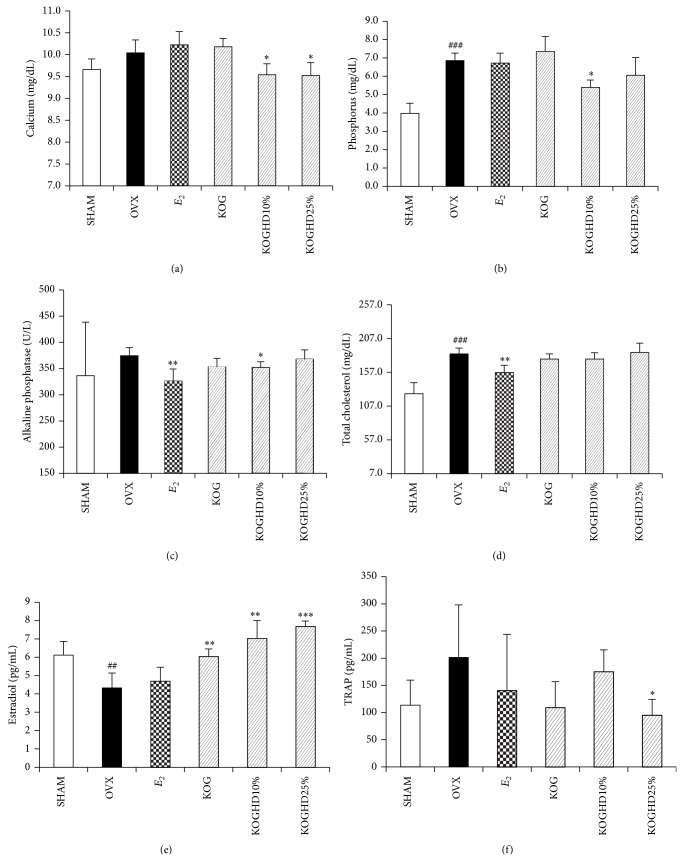
Effect of KOG, KOGHD10%, and KOGHD25% on serum biochemical markers. In control, SHAM-operated mice and OVX mice with or without the administration of three agents (185 mg/kg/day, p.o) for 6 weeks, serum (a) calcium, (b) phosphorus, (c) ALP, and (d) TCHO were determined by using a diagnostic slide. (e) Estradiol and (f) TRAP were analysed by ELISA kit as described in materials and methods. Data are expressed as means ± SD, *n* = 5. ^*^ *P* < 0.05, ^**^ *P* < 0.01, and ^***^ *P* < 0.001 versus OVX group; ^#^ *P* < 0.05, ^##^ *P* < 0.01, and ^###^ *P* < 0.001 versus SHAM group.

After 6 weeks of oral administration of KOG, KOGHD10%, and KOGHD25%, biochemical markers (calcium, phosphorus, alkaline phosphatase, and total cholesterol) were measured from serum. As compared with the OVX group (9.64 mg/dL ± 0.167), serum calcium levels were lower in the KOGHD10% and KOGHD25% groups (10.08 mg/dL ± 0.363, *P* < 0.05 versus 9.88 mg/dL ± 0.522, *P* < 0.05). Serum calcium secretion in the OVX group was greater than in the SHAM group (10.04 mg/dL ± 0.241), suggesting active resorption in bones (Figure 5(a)). Serum phosphorus levels were lower in the KOGHD10% (5.4 mg/dL ± 0.394, *P* < 0.05) and KOGHD25% (6.06 mg/dL ± 0.961) groups than in the OVX group (6.86 mg/dL ± 0.404) (Figure 5(b)), and serum ALP levels were lower in the KOGHD10% (351.6 mg/dL ± 11.696, *P* < 0.01) group than in the OVX group (374.6 mg/dL ± 15.469) (Figure 5(c)). No significant differences in serum TCHO levels were found between the OVX and the KOG, KOGHD10%, and KOGHD25% (Figure 5(d)). Serum *E*_2_ concentrations in the OVX group (4.325 pg/mL ± 0.808) were lower than in the SHAM group (6.115 pg/mL ± 0.747), and *E*_2_ levels were concentration dependently increased in KOG (6.048 pg/mL ± 0.406), KOGHD10% (7.036 pg/mL ± 0.973), and KOGHD25% (7.685 pg/mL ± 0.287) groups as compared with the OVX group (Figure 5(e)). Serum TRAP concentrations in the OVX group were increased compared to that in SHAM group and KOGHD25% treatment of OVX mice significantly reduced serum TRAP concentrations (Figure 5(f)).

### 3.6. Effect of KOG, KOGHD10%, and of KOGHD25% on Immune Cells Distribution in the Spleen and Thymus

**Figure 6:**
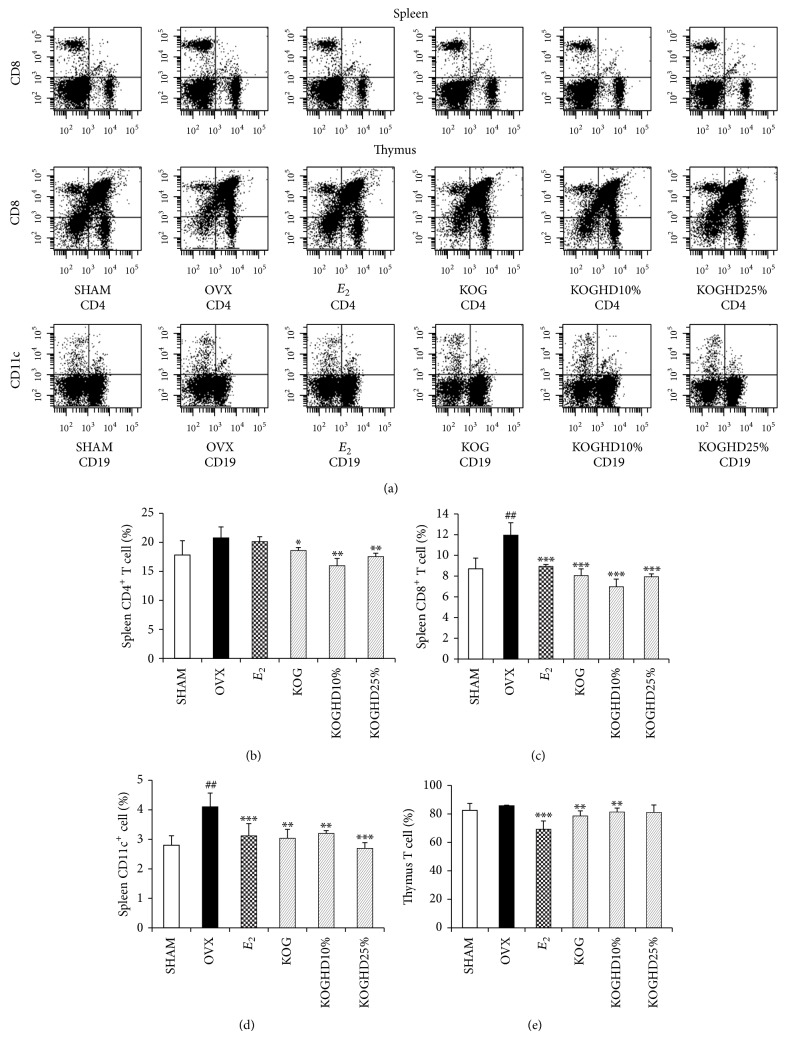
Expression of T cell and dendritic cell on splenic or thymic lymphocytes. They were treated with vehicle, sample (185 mg/kg/day, p.o) for 6 weeks. Data are expressed as means ± SD, *n* = 5. ^*^ *P* < 0.05, ^**^ *P* < 0.01, and ^***^ *P* < 0.001 versus OVX group; ^#^ *P* < 0.05, ^##^ *P* < 0.01, and ^###^ *P* < 0.001 versus SHAM group.

We examined the effects of the oral administration of KOG, KOGHD10%, and KOGHD25% on lymphocyte subsets in spleen and thymus by flow cytometry (Figure 6(a)). Immune cells in the OVX group showed higher expressions of CD4^+^ T cell, CD8^+^ T cell, and CD11c^+^ cell than those in the SHAM group. CD19 (B cell) population was not changed between the SHAM and OVX group (data not show). KOG, KOGHD10%, or KOGHD25% treatment reduced the population of CD4^+^, CD8^+^, and CD11c^+^ cells, confirming the population of T cells (Double positive) in the thymus. The cell population is greater in the KOG and KOGHD10% groups than in the OVX group (Figures 6(b), 6(c), and 6(d)).

### 3.7. Effects of KOG, KOGHD10%, and of KOGHD25% on the Microarchitecture of Bone

**Figure 7:**
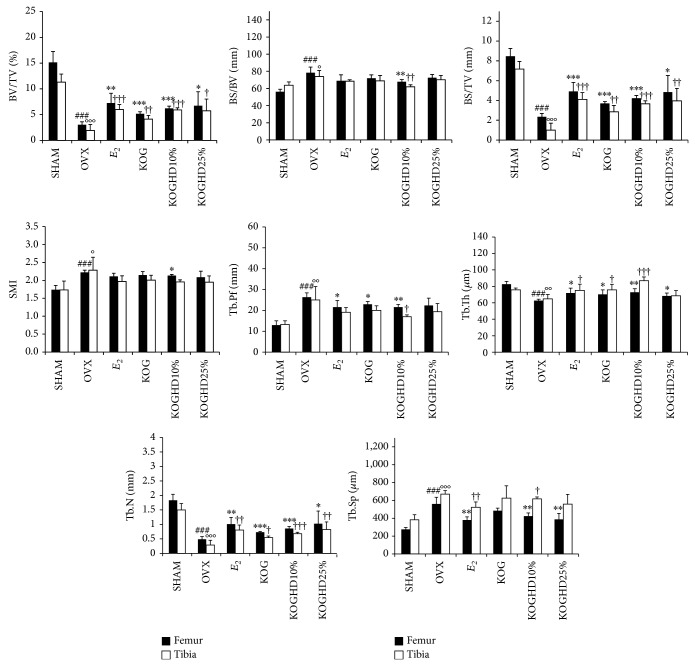
Effect of KOG, KOGHD10%, and KOGHD25% on trabecular morphometric parameters in proximal tibia and distal femur of OVX mice. Mice were treated with vehicle, HD (185 mg/kg/day, p.o) for 6 weeks. bone volume/tissue volume (BV/TV), bone surface/tissue volume (BS/TV), bone surface/Bone volume (BS/BV), structure model index (SMI), trabecular bone pattern factor (Tb.Pf), trabecular thickness (Tb.Th), trabecular number (Tb.N), and trabecular separation (Tb.Sp) as analyzed with micro-CT Skyscan CTAn software. Data are expressed as mean SD (*n* = 5). ^*^ *P* < 0.05, ^**^ *P* < 0.01, and ^***^ *P* < 0.001 versus OVX group (femur); ^#^ *P* < 0.05, ^##^ *P* < 0.01, and ^###^ *P* < 0.001 versus SHAM group (femur); ^†^ *P* < 0.05, ^††^ *P* < 0.01, and ^†††^ *P* < 0.001 versus OVX group (tibia); °*P* < 0.05, °°*P* < 0.01, and °°°*P* < 0.001 versus SHAM group (tibia).

To determine whether the oral administration of KOG, KOGHD10%, and KOGHD25% constitutes feasible treatments for osteoporosis, bone masses were analyzed by micro-CT. In proximal tibial metaphyses, micro-CT demonstrated that ovariectomy reduced bone volume density. The SHAM group (11.317 ± 1.569) had a greater BV/TV (bone volume/total volume) than the OVX group (1.915 ± 1.166). BV/TV was also greater in the KOG, KOGHD10%, and KOGHD25% groups (4.125 ± 0.736 versus 5.903 ± 0.43 versus 5.742 ± 2.281) than that in the OVX group. BS/TV (bone surface/total volume) in the SHAM group (7.174 ± 0.763) was higher than that in the OVX group (1 ± 0.696) and dose-dependently increased in the KOG, KOGHD10%, and KOGHD25% groups (2.852 ± 0.634 versus 3.664 ± 0.297 versus 3.948 ± 1.271). The BS/BV (bone surface/bone volume) in the only KOGHD10% group (62.054 ± 2.343) was lower than that in the OVX group (74 ± 7.015). Furthermore, SMI (structure model index) in the SHAM group (1.733 ± 0.249) was lower than in the OVX group (2.282 ± 0.364). However, there was no difference observed between the OVX group and the other study groups. Tb.Pf (trabecular bone pattern factor) in the OVX group (25 ± 6.421) was greater than that in the SHAM group (13.282 ± 1.701) and Tb.Pf in the KOG and KOGHD10% (20.042 ± 2.172 and 16.990 ± 0.858, resp.) groups were lower than that in the OVX group. The SHAM group (75.685 ± 2.314) had a greater Tb.Th (trabecular thickess) than the OVX group (64.879 ± 5.311), and the KOG and KOGHD10% groups (75.737 ± 6.771 and 86.888 ± 4.576, resp.) had greater Tb.Th values than the OVX group. Tb.N (trabecular number) was lower in the OVX group (0.288 ± 0.162) than in the SHAM group (1.498 ± 0.219). Mean Tb.N values in the KOG, KOGHD10%, and KOGHD25% groups (0.542 ± 0.058 versus 0.678 ± 0.049 versus 0.824 ± 0.259) showed concentration dependent increases versus the OVX group. Tb.Sp (trabecular separation) was greater in the OVX group (670.879 ± 39.15) than in the SHAM group (383.202 ± 57.195) and in the KOGHD10% group (617.536 ± 20.631). In distal femoral metaphyses, BV/TV was lower in the OVX group (3.024 ± 0.582) than in the SHAM group (15.138 ± 2.113). The KOG, KOGHD10%, and KOGHD25% groups (5.153 ± 0.406 versus 6.218 ± 0.421 versus 6.745 ± 2.709) showed concentration-dependent increases as compared with the OVX group. The SHAM group (8.466 ± 0.794) had a higher BS/TV than the OVX group (2.34 ± 0.348), and mean BS/TV values in the KOG, KOGHD10%, and KOGHD25% groups (3.694 ± 0.186 versus 4.212 ± 0.291 versus 4.824 ± 1.698) showed concentration-dependent increases. BS/BV and SMI values of the KOGHD10% group (67.798 ± 2.612, 2.132 ± 0.031), but not those of the KOG and KOGHD25% groups, were lower than in the OVX group (78.408 ± 6.675, 2.221 ± 0.062). The SHAM group (82.644 ± 3.451, 1.83 ± 0.21) showed an evaluation in the Tb.Th and Th.N as compared to OVX group (62.708 ± 1.649, 0.484 ± 0.093). Increases in Tb.Th and Tb.N were observed in the KOG, KOGHD10%, and KOGHD25% groups (70.33 ± 5.387 versus 72.872 ± 4.296 versus 68.351 ± 3.5, 0.732 ± 0.026 versus 0.856 ± 0.072 versus 1.024 ± 0.434). Tb.Pf and Tb.Sp were higher in the OVX group (26.328 ± 2.088, 560.216 ± 74.548) than in the SHAM group (12.924 ± 2.078, 274.901 ± 22.313). The KOG and KOGHD10% groups (21.554 ± 1.264, 423.427 ± 36.517) had lower Tb.Pf and Tb.Sp values than the OVX group (Figure 7). Three-dimensional (3D) images of proximal tibiae and distal femoral metaphyses showed that bone loss in OVX mice was dramatically inhibited by the oral administration of KOG, KOGHD10%, and KOGHD25% (Figure 9).

### 3.8. Trabecular Bone Mineral Density of Femur and Tibia

**Figure 8:**
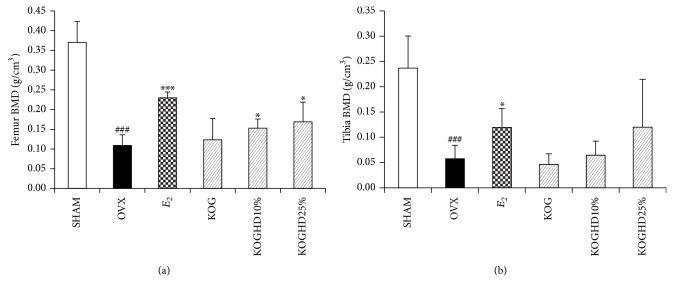
Effect of KOG, KOGHD10%, and KOGHD25% on BMD of (a) femur and (b) tibia of OVX mice. Data are expressed as means ± SD, *n* = 5. ^*^ *P* < 0.05, ^**^ *P* < 0.01, and ^***^ *P* < 0.001 versus OVX group; ^#^ *P* < 0.05, ^##^ *P* < 0.01, and ^###^ *P* < 0.001 versus SHAM group.

**Figure 9:**
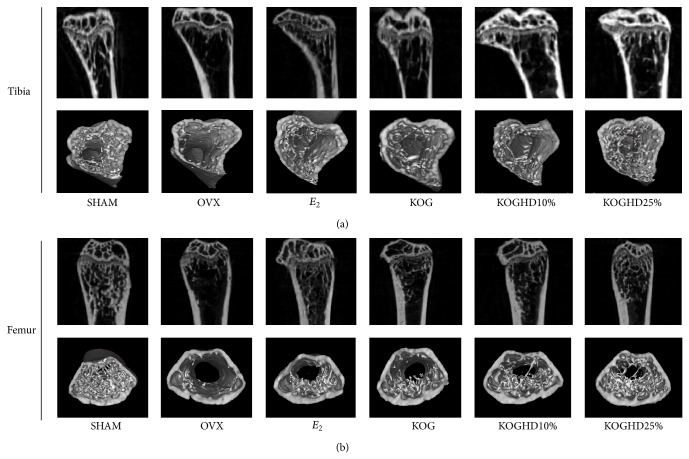
Analysis of microcomputed tomography in the region of the proximal tibia and distal femur after sacrifice. Representative 3D images of (a) tibia and (b) femur.

The BMD of the tibia and femur is presented in Figure 8. These results demonstrate that OVX significantly decreased the BMD by 26.12% in the femur and by 17.94% in the tibia compared to the SHAM group (both *P* < 0.001). Compared to the OVX group, KOGHD treatment obviously prevented the BMD decrease in OVX-induced femur (*P* < 0.05) in a dose independent manner. *E*_2_ also significantly increased the BMD of the femur and tibia. However, KOGHD treatment did not significantly prevent the BMD decrease in OVX-induced tibia.

### 3.9. Histological Analysis of Bone

**Figure 10:**
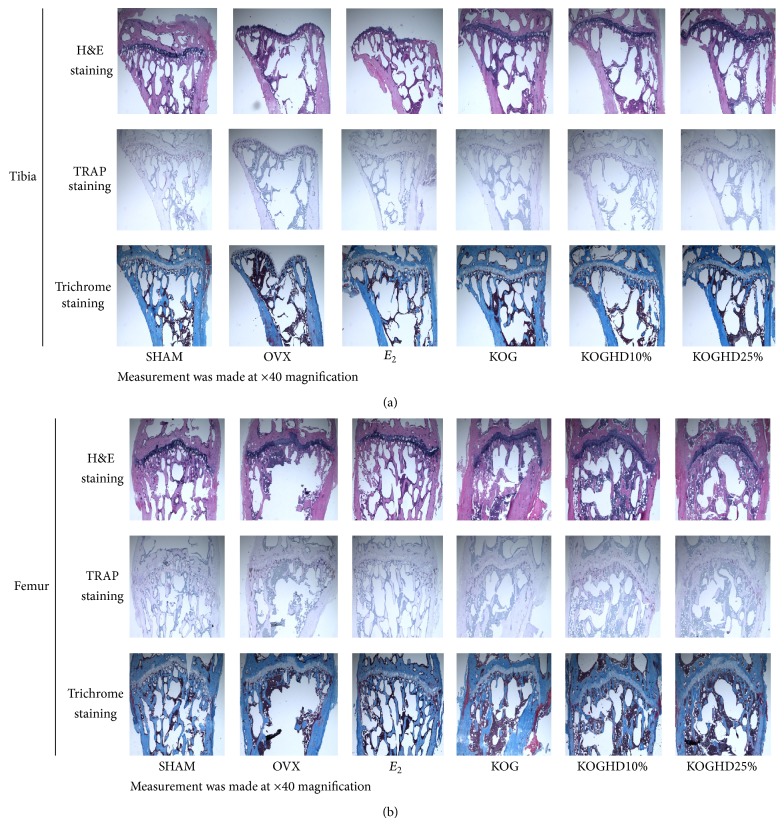
Histological analysis of (a) proximal tibia and (b) distal femur with H&E, TRAP, and Masson's trichrome staining. Measurement was made at ×40 magnification.

Histomorphologies of tibiae and femurs were investigated with H&E, TRAP, and Masson trichrome staining. The proximal head of tibiae, distal femurs, trabecular bone of tibiae, and femurs in the OVX group were thinner, smaller, and wider interval and showed less connectivity than the SHAM group, whereas the opposite was observed in the KOG, KOGHD10%, and KOGHD25% groups. That is to say, bones in the KOG, KOGHD10%, and KOGHD25% groups were more compact than bones in the OVX group (Figure 10).

## 4. Discussion

Postmenopausal osteoporosis has become a social problem requiring appropriate therapeutic and preventive strategies. Ovariectomized osteoporosis mouse models can be useful for drug development and in the skeletal research field [19]. Although hormone replacement therapy presents the risk of breast cancer and venous thromboembolism, many women are still prescribed estrogen therapy for postmenopausal symptoms [20, 21]. Tamoxifen is one of the selective estrogenic receptor modulators (SERMs) and is widely used for the prevention and treatment of breast cancer. However, the prolonged use of tamoxifen increases the risk of endometrial cancer. More recently, another SERM, raloxifene, was found not to have ER agonist properties in the uterus [22], and thus there is need to find more SERM-like substances with reduced side effects on the breast and uterus.

Estrogen therapy after the menopause increases the risk of breast cancer. However, phytoestrogens, which are widely distributed in plants, are structurally similar to mammalian estrogens and can thus bind weakly to estrogen receptors [23]. Epidemiologic data demonstrate that the consumption of phytoestrogens decreases the incidence of breast cancer [24]. Furthermore, the fruit extracts of* Hovenia dulcis* (HD) activate the Wnt/B-catenin pathway and modulate osteoblast differentiation and bone mass* in vitro* and* in vivo*, respectively [25]. RANKL-induced the formation of osteoclast was suppressed by KOG mixed with extract of HD than only KOG treatment* in vitro* experiment. Also, micro-CT parameters of the distal femur and proximal tibia showed that HD reduced bone deterioration in OVX mice. Although BMD was not significantly different in the HD and OVX groups, treatment with HD increased BMD in femur and tibia. In the 2D images showed that HD protective trabecular bone loss in OVX mice (Figure 2(c)). However, the protective effect of HD against bone loss is slight. Therefore, we hypothesized that KOG mixed with extract of HD may have a synergic effect in preventing bone loss more than only KOG treatment.

In the present study, KOG, KOGHD10%, and KOGHD25% were orally administered to ovariectomized mice for 6 weeks and were investigated with respect to the inhibition of bone loss. Some studies have reported that estradiol could be useful of further investigation of physiological and pharmacological effects of estrogen and other agents on bone* in vivo* [26]. Thus, we used water soluble *β*-estradiol (0.03 *μ*g/head/day, s.c, Sigma-Aldrich, St. Louis, MO, USA) as the positive control.

In this study, an OVX mice model was used to evaluate the efficacies of KOG and KOGHD for the treatment of postmenopausal osteoporosis. The success of OVX was confirmed by marked atrophy of uteri and dramatic decreases in uterine weights (estrogen directly influences uterine weights). Moreover, ovariectomy also increases body, spleen, and thymic weights [27, 28]. No dose of three groups (KOG, KOGHD10%, and KOGHD25%) prevented the body weight and thymus weight gain induced by estrogen deficiency. However, the uterine weight loss and spleen weight gain associated with ovariectomy were prevented by treatment with KOGHD25%.

Loss of bone mass and the deterioration of bone microstructure have been linked to an imbalance between bone formation and bone resorption, and thus biochemical markers of bone turnover have been widely used as research tools to measure the effects of various drugs on bone remodeling [29]. The oral administration of KOGHD suppressed the loss of calcium and phosphorus from serum, indicating that it acts to maintain the physiological calcium and phosphorus level. Also, this inhibits activation of TRAP, a marker of osteoclast differentiation [30]. Some studies have shown that natural products and dietary components, such as phytoestrogens, have protective effects on bone remodeling by inhibiting bone resorption [31]. Serum estradiol decreased in OVX mice, but KOGHD25% reduced this effect.

Some studies have reported that weights and lengths of tibiae and femurs in ovariectomized mice are lower than in sham operated mice [32]. In the present study, although bone weights were not significantly different in the SHAM and OVX groups, treatments with KOGHD10% and KOGHD25% significantly increased tibia and femur weight versus OVX mice. In the growth hormone treated animal models, cancellous bone mass is increased. Thus, KOGHD may have effects similar to growth hormone stimulatory effects on bone weights growth [33].

In a study on bone loss induced by ovariectomy, T cells were found to play an important role in the stimulation of osteoclastogenesis induced by postmenopausal osteoporosis in woman [34]. Estrogen deficiency leads to an increase in the immune function, which augments T cell activation [35]. Balakrishnan et al. reported that lymphocytes from mice in an OVX group showed higher expressions of CD3, CD4, and CD8 than sham-operated controls [36]. Dendritic cells (DCs) are the most powerful antigen-presenting cells, and OVX induces the activation of both CD4^+^ and CD8^+^ cells by antigen presentation of DCs. Grassi et al. reported that the DC population in ovariectomized mice bone marrow was increased as compared with SHAM mice [37]. This was confirmed in the present study as ovariectomy increased the expressions of CD4^+^, CD8^+^, and CD11c^+^ on leukocytes. Furthermore, ovariectomized mice administered KOG, KOGHD10%, or KOGHD25% for 6 weeks reduced expressions of CD4^+^, CD8^+^, and CD11c^+^ on leukocytes.

Some studies have shown that ovariectomy leads to significant deterioration of trabecular bone microarchitecture and bone loss in mice [38]. In the present study, micro-CT parameters of the distal femurs and proximal tibiae showed that the oral administration of the three agents for 6 weeks reduced bone deterioration in OVX mice. In addition, KOG mixed with HD had a more positive effect on trabecular bone microarchitecture than KOG alone.

Hormone deficiency is known to impair cancellous metaphyseal bone and reduce BMD in humans and animals [39]. Bone mineral density (BMD) is considered to be the standard measure for the diagnosis of osteoporosis and postmenopausal bone loss is characterized by a decrease in BMD [40]. The results in the present study showed that OVX reduced BMD in the femur, which are rich in cancellous bone, while treatment with KOGHD prevented the decreases in BMD.

Some authors have reported that trabecular in 3D images of the distal femurs of OVX mice were lower than in SHAM mice [41, 42]. Our 3D images showed bone loss in OVX mice was dramatically inhibited by the oral administration of KOGHD. In addition, we performed histological analysis of proximal tibiae and distal femurs and found treatment with KOGHD, more so than with KOG, inhibited trabecular bone loss (H&E staining), and osteoclastic bone resorption by suppressing TRAP activity (TRAP staining). This finding indicates that KOGHD increased trabeculation and maintained the primary cancellous bone of tibiae and femurs.

Osteoclasts are the only cell type capable of resorbing bone, and thus enhancement of osteoclast activity plays an important role in bone loss [43]. Monocytes/macrophage ligand cells in bone marrow differentiate into mature osteoclasts in the presence of M-CSF and RANKL [44] and in the present study shows that the inhibitory effects of KOGHD on RANKL-induced osteoclast differentiation were not associated with a cytotoxic effect. Therefore, the present study demonstrates that KOGHD inhibits ovariectomy-induced bone loss by reducing osteoclast differentiation without cytotoxic effect.

## 5. Conclusions

Our study showed that daily oral administration of KOGHD over a 6-week period prevents estrogen deficiency-induced bone loss, inhibits the deterioration of trabecular microarchitecture, decreases augmented T lymphocyte numbers as well as dendritic cell, maintains bone turnover rate, and suppressed decrease of the uterus weight. Moreover, KOGHD significantly inhibit the differentiation of osteoclasts. Collectively, our* in vivo* and* in vitro* results suggest that new oriental medicine preparation (KOG mixed with HD water extract) may have therapeutic potential for the treatment of postmenopausal osteoporosis.

## Figures and Tables

**Table 1:** Thymus and spleen weight in C3H/HeN mice.

Parameter	SHAM	OVX	*E*_2_	KOG	KOGHD15%	KOGHD25%
Thymus WT (mg)	25.6 ± 2.55	28.48 ± 3.53	22.84 ± 5.16	24 ± 3.75	27.52 ± 2.04	26.68 ± 1.84
Spleen WT (mg)	74.9 ± 3.6	82.34 ± 3.69^#^	79.8 ± 4.76	76.7 ± 4.78	76.62 ± 4.31	72.58 ± 6.75^*^

KOGHD25% treatment decreases OVX-induced increase in spleen weight. ^*^ *P* < 0.05 versus the OVX group; ^#^ *P* < 0.05 versus SHAM group.

**Table 2:** Effect of KOG, KOGHD10%, and KOGHD25% on anatomical properties in bone of OVX mice for 6 weeks.

Group	Tibia		Femur	
	Weight (mg)	Length (mm)	Weight (g)	Length (mm)
SHAM	45.4 ± 2.8	18.498 ± 0.14	58.0 ± 4.3	15.734 ± 0.144
OVX	44.3 ± 3.0	18.675 ± 0.39	57.9 ± 3.9	15.917 ± 0.453
KOG	46.6 ± 2.9	18.833 ± 0.296	58.3 ± 3.3	16.068 ± 0.137
KOGHD10%	48.3 ± 2.1^**^	18.62 ± 0.107	62.6 ± 1.8^**^	16.008 ± 0.349
KOGHD25%	49.6 ± 1.7^**^	18.652 ± 0.37	65.1 ± 2.4^**^	15.941 ± 0.174

They were treated with vehicle, sample (185 mg/kg/day, p.o) for 6 weeks. ^**^ *P* < 0.01 versus OVX group at corresponding parameters.
